# Gold Nanoparticles on Mesoporous SiO_2_-Coated Magnetic Fe_3_O_4_ Spheres: A Magnetically Separatable Catalyst with Good Thermal Stability

**DOI:** 10.3390/molecules181114258

**Published:** 2013-11-18

**Authors:** Huan Liu, Chao Lin, Zhen Ma, Hongbo Yu, Shenghu Zhou

**Affiliations:** 1 Research Institute of Applied Catalysis, Shanghai Institute of Technology, Shanghai 201418, China; 2 Department of Physics, Faculty of Science, Ningbo University, Ningbo 315211, China; 3 Shanghai Key Laboratory of Atmospheric Particle Pollution and Prevention (LAP^3^), Department of Environmental Science and Engineering, Fudan University, Shanghai 200433, China; 4 Ningbo Institute of Materials Engineering and Technology, Chinese Academy of Sciences, Ningbo 315201, China

**Keywords:** solvothermal method, magnetic Fe_3_O_4_ spheres, *p*-nitrophenol, gold catalysis

## Abstract

Fe_3_O_4_ spheres with an average size of 273 nm were prepared in the presence of CTAB by a solvothermal method. The spheres were modified by a thin layer of SiO_2_, and then coated by mesoporous SiO_2_ (m-SiO_2_) films, by using TEOS as a precursor and CTAB as a soft template. The resulting m-SiO_2_/Fe_3_O_4_ spheres, with an average particle size of 320 nm, a high surface area (656 m^2^/g), and ordered nanopores (average pore size 2.5 nm), were loaded with gold nanoparticles (average size 3.3 nm). The presence of m-SiO_2_ coating could stabilize gold nanoparticles against sintering at 500 °C. The material showed better performance than a conventional Au/SiO_2_ catalyst in catalytic reduction of *p*-nitrophenol with NaBH_4_. It can be separated from the reaction mixture by a magnet and be recycled without obvious loss of catalytic activity. Relevant characterization by XRD, TEM, N_2_ adsorption-desorption, and magnetic measurements were conducted.

## 1. Introduction

Gold was initially regarded as useless in catalysis, until Haruta and co-workers found that small gold nanoparticles supported on some reducible oxide supports can be highly active for CO oxidation [1,2,3]. This finding triggered a great deal of interest in exploring the application of gold catalysts in other reactions [4,5,6], such as organic catalysis [7,8,9,10,11]. Most of the heterogeneous gold catalysts reported so far involve oxide supports such as TiO_2_, ZrO_2_, Fe_2_O_3_, CeO_2_, Al_2_O_3_, and SiO_2_. These oxide supports are not magnetic, thus making the supported gold catalysts difficult to separate after conducting organic reactions. In addition, many gold catalysts tend to sinter under elevated temperatures due to the low melting points of gold nanoparticles. The sintering can occur even under mild reaction conditions in organic catalysis. Therefore, for the sake of practical applications, it is desirable to design magnetically separable gold catalysts with good thermal stability.

Fe_3_O_4_ is a magnetic oxide. It can be used for designing magnetically separable catalysts and other functional materials [12,13,14,15,16,17,18,19,20]. For instance, Yin and co-workers prepared SiO_2_/Au/Fe_3_O_4_ catalysts by coating an Au/Fe_3_O_4_ catalyst with a SiO_2_ matrix, followed by controlled etching [21]. That way, the gold nanoparticles were protected by the SiO_2_ shell, and the SiO_2_ shell was porous, allowing for the diffusion of reactants and products. Alternatively, Zhao and co-workers prepared SiO_2_/Au/Fe_3_O_4_ catalysts by assembling a porous SiO_2_ shell on top of an Au/Fe_3_O_4_ catalyst, with the aid of a soft template [22]. The resulting SiO_2_/Au/Fe_3_O_4_ catalyst has enhanced stability against sintering. These SiO_2_/Au/Fe_3_O_4_ catalysts are particularly useful in organic catalysis, because they can be separated from the liquid phase after reaction by simply using a magnet.

**Scheme 1:**
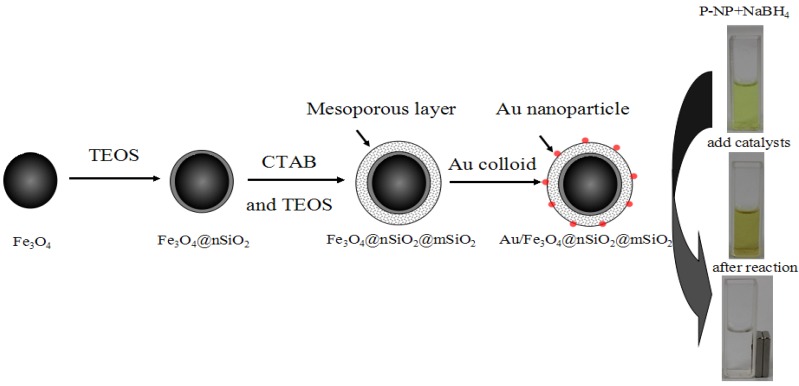
Synthesis of Au/m-SiO_2_/Fe_3_O_4_ that can be separated by a magnet.

Here we prepare another catalyst, Au/m-SiO_2_/Fe_3_O_4_ (Scheme 1). First, magnetic Fe_3_O_4_ particles were prepared by a solvothermal method. The particles were treated by a small amount of TEOS in the absence of a soft template (CTAB), and subsequently coated by mesoporous SiO_2_ (m-SiO_2_) films with the aid of the soft template. Gold nanoparticles were then deposited onto the m-SiO_2_-coated Fe_3_O_4_ support. The resulting catalyst is magnetically separable, thermally stable, and shows better catalytic activity than Au/SiO_2_ in the catalytic reduction of *p*-nitrophenol with NaBH_4_.

## 2. Results and Discussion

**Figure 1:**
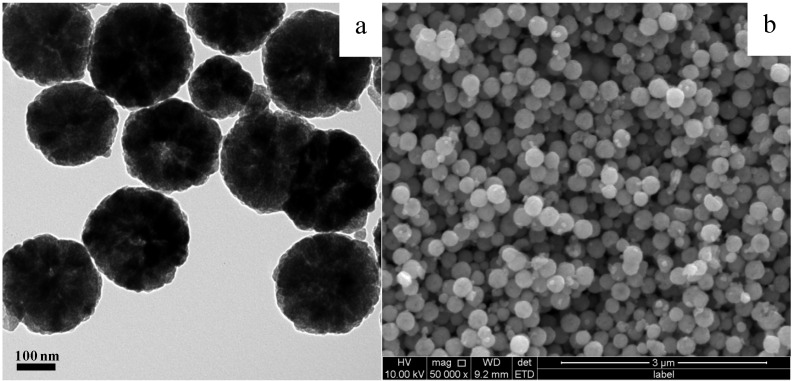
TEM (**a**) and SEM (**b**) images of Fe_3_O_4_ spheres.

Many references have reported the synthesis of Fe_3_O_4_ spheres in the presence of a protecting agent [15,23,24,25]. The formation of Fe_3_O_4_ spheres generally involves nanocrystal nucleation, crystal growth, and self-assembly [24]. Our work used CTAB as a protecting agent. The synthesized Fe_3_O_4_ particles are spherical, as seen from the TEM and SEM images in Figure 1. The particle size distribution obtained from TEM analysis of a number of particles is shown in Figure S1 in the Supplementary Materials. The average particle size is 273 nm.

**Figure 2:**
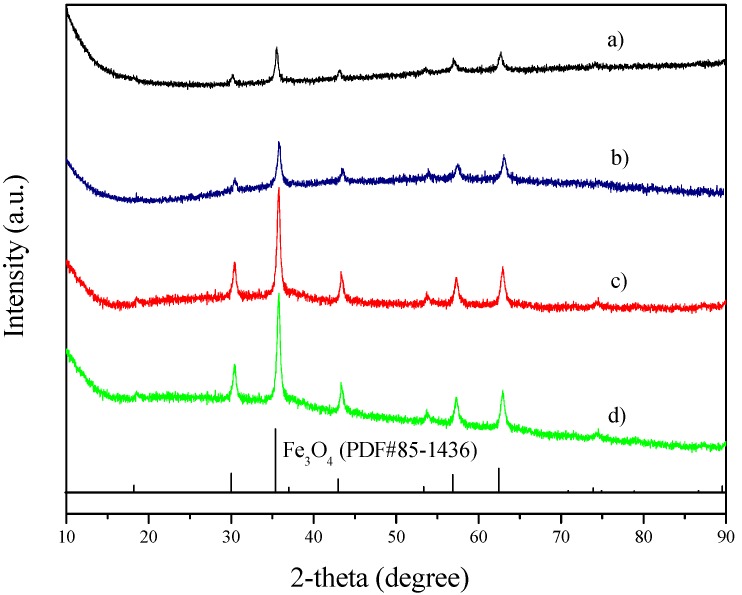
XRD patterns of (**a**) Fe_3_O_4_ spheres; (**b**) m-SiO_2_/Fe_3_O_4_ without calcination; (**c**) m-SiO_2_/Fe_3_O_4_ calcined at 500 °C to remove the soft template; (**d**) Au/m-SiO_2_/Fe_3_O_4_.

The Fe_3_O_4_ sample has characteristic XRD peaks at 2θ = 30.2°, 35.6°, 43.2°, 53.6°, 57.2°, 62.8°, and 74.2° (Figure 2a), corresponding to the cubic phase of Fe_3_O_4_.

**Figure 3:**
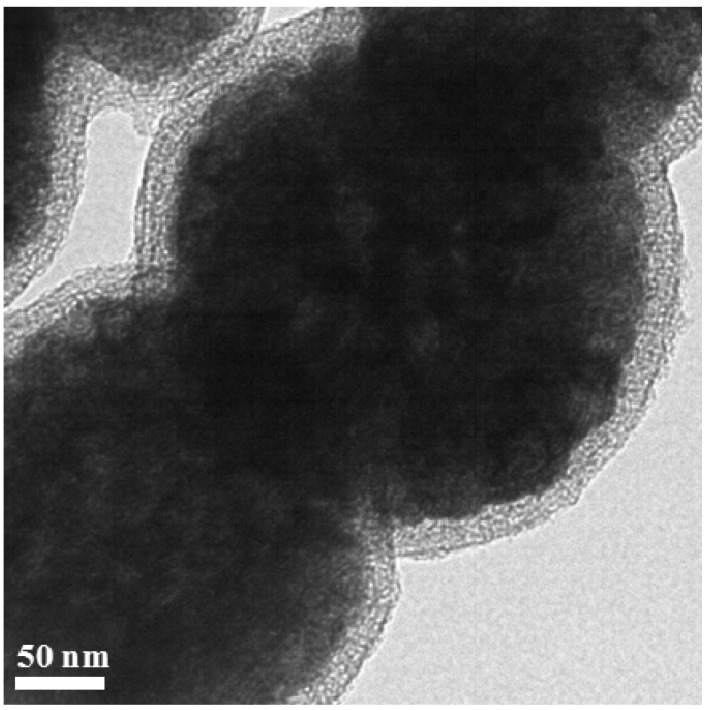
TEM image of m-SiO_2_/Fe_3_O_4_.

To synthesize m-SiO_2_/Fe_3_O_4_, the Fe_3_O_4_ particles were first treated by a small amount of TEOS, resulting in the modification of Fe_3_O_4_ particles with a thin layer of SiO_2_ (see Figure S2 in the Supplementary Materials). Then the SiO_2_-modified Fe_3_O_4_ particles were treated with more TEOS in the presence of a soft template (CTAB), followed by calcination to remove the soft template, resulting in the formation of m-SiO_2_/Fe_3_O_4_. The average size of m-SiO_2_/Fe_3_O_4_ particles is about 320 nm, as seen from the TEM image in Figure 3. Figure 3 also shows that the thickness of the SiO_2_ layer is about 27 nm, and the SiO_2_ layer is porous. Figure S3 shows more TEM images of the sample.

**Figure 4:**
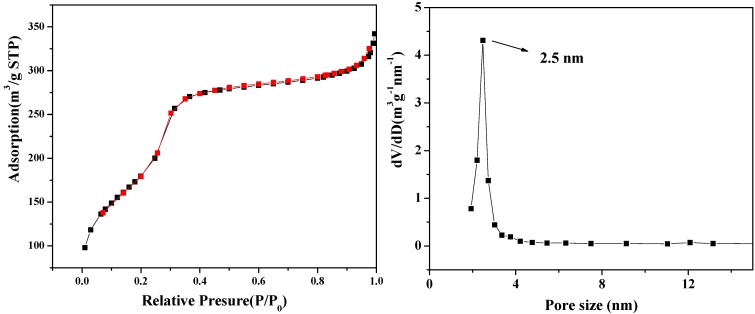
The nitrogen adsorption-desorption isotherms and pore size distribution of m-SiO_2_/Fe_3_O_4_.

The material has a high surface area of 656 m^2^/g and an average pore size of 2.5 nm (Figure 4). For comparison, the surface area of Fe_3_O_4_ particles is only 30 m^2^/g.

The SiO_2_ coating is amorphous, as shown by the XRD data (Figure 2b,c). Note that the material was calcined at 500 °C to remove the soft template and create mesopores. This calcination process increases the crystallinity of the Fe_3_O_4_ particles, as seen from the sharper peaks in Figure 2c.

**Figure 5:**
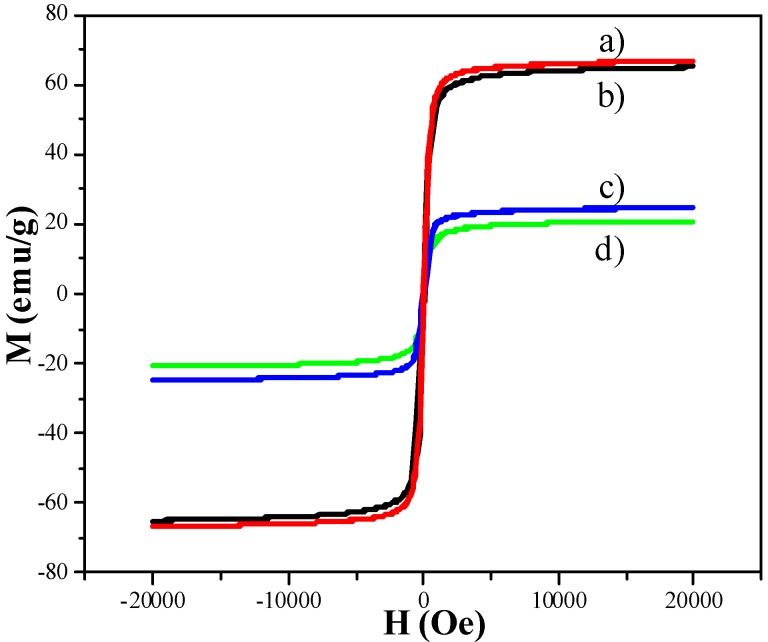
Hysteresis loops of samples at room temperature: (**a**) Fe_3_O_4_ particles; (**b**) thin SiO_2_-modified Fe_3_O_4_; (**c**) m-SiO_2_/Fe_3_O_4_; (**d**) Au/m-SiO_2_/Fe_3_O_4_.

Figure 5 shows the magnetization curves of samples. The saturated susceptibility of Fe_3_O_4_ spheres is 65.0 emu/g. The modification of Fe_3_O_4_ spheres by a thin layer of SiO_2_ leads to a negligible decrease in saturated susceptibility. The saturated susceptibility of m-SiO_2_/Fe_3_O_4_ is 24.7 emu/g. The loading of gold nanoparticles onto m-SiO_2_/Fe_3_O_4_ leads to a negligible decrease in saturated susceptibility.

**Figure 6:**
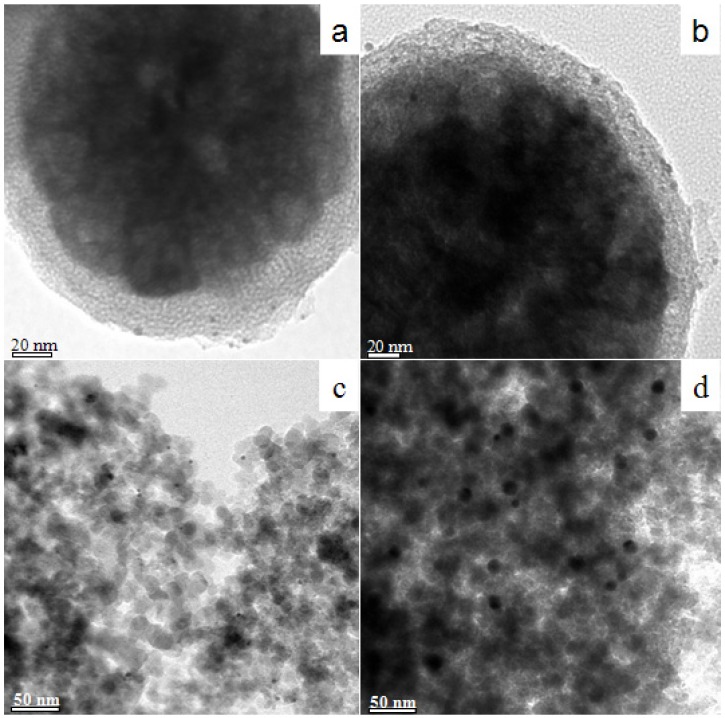
TEM images of as-synthesized Au/m-SiO_2_/Fe_3_O_4_ (**a**), Au/m-SiO_2_/Fe_3_O_4_ calcined at 500 °C (**b**), as-synthesized Au/SiO_2_ (**c**) and Au/SiO_2_ calcined at 500 °C (**d**).

Thermal stability is an important factor for practical applications of gold catalysts. Figure 6 shows the TEM images of Au/m-SiO_2_/Fe_3_O_4_ samples as prepared and calcined at 500 °C. For the as-prepared Au/m-SiO_2_/Fe_3_O_4_, the gold nanoparticles are highly dispersed, with an average particle size of 3.3 nm. The particle size distribution is shown in Figure S4. When the Au/m-SiO_2_/Fe_3_O_4_ is calcined at 500 °C for 2 h, the average gold particle size increases only slightly to 3.8 nm. On the other hand, the average size of gold nanoparticles in Au/SiO_2_ increases from 3.4 nm to 13.4 nm after calcination at 500 °C. Although the average size of gold nanoparticles (3.3 nm) is larger than the average pore size of the mesoporous SiO_2_ coating, the presence of mesoporous SiO_2_ still mitigates the sintering of gold nanoparticles.

**Figure 7:**
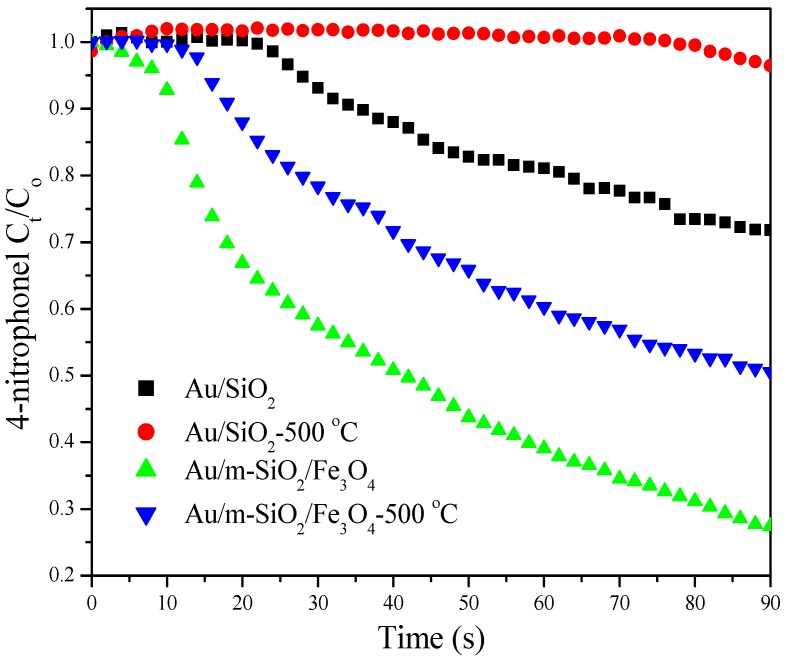
Decrease in *p*-nitrophenol’s relative concentration during the hydrogenation reaction using different catalysts.

Catalytic reduction of *p*-nitrophenol by NaBH_4_ was chosen to compare the performance of different gold catalysts. The reaction was carried out in a cuvette (rather than a round-bottom flask reported previously [26]) to allow for *in situ* monitoring. The reaction progress was followed by UV-Vis as the peak around 400 nm corresponds to the absorption of *p*-nitrophenol. Figure 7 shows the decreases in *p*-nitrophenol concentrations as the reaction proceeds. A faster decrease indicates higher catalytic activity. The conversions of *p*-nitrophenol on Au/m-SiO_2_/Fe_3_O_4_ and Au/SiO_2_ after 90 s reaction are 72.5% and 28.2%, respectively. After calcination at 500 °C, these catalysts show conversions of 49.4% and 4.5%, respectively. The catalysis data (also seen in Figure S5 and Table S1) again show the advantage of using a mesoporous SiO_2_ coating. As the catalytic activity drops greatly when the size of gold nanoparticles is increased [26], the low activity of the sintered Au/SiO_2_ catalyst is justified.

**Figure 8:**
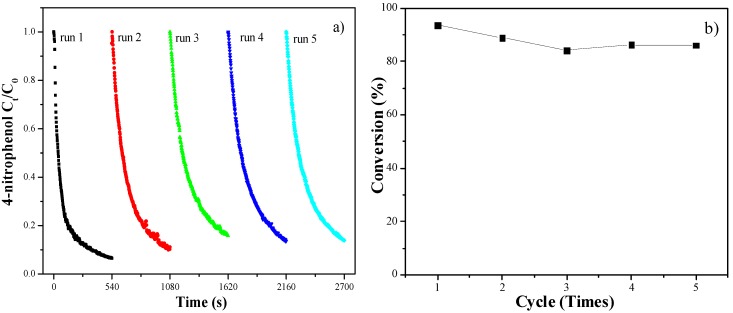
Performance of Au/m-SiO_2_/Fe_3_O_4_ in repeated runs, after recycling of the catalyst.

The catalyst recyclability was studied by testing the activity of Au/m-SiO_2_/Fe_3_O_4_ after separation using a magnet (see the photos in Scheme 1). No additional catalyst was added into the liquid phase. As shown in Figure 8, Au/m-SiO_2_/Fe_3_O_4_ shows stable activity in repeated runs.

## 3. Experimental

### 3.1. Chemicals

All chemicals were used as received. Anhydrous FeCl_3_, sodium acetate, ethylene glycol, aqueous ammonia, polyvinylprrolidone (PVP, MW-58000), *p*-nitrophenol was purchased from Sinopharm Chemical Reagent Co. (Shanghai, China). Tetraethoxysilane (TEOS), hexadecyl trimethyl ammonium bromide (CTAB), HAuCl_4_·4H_2_O and NaBH_4_ were purchased from Aladdin Co. (Shanghai, China).

### 3.2. Catalyst Preparation

#### 3.2.1. Preparation of Fe_3_O_4_ Spheres

Anhydrous FeCl_3_ (0.54 g), CTAB (0.2 g) and sodium acetate (2.5 g) were dissolved in ethylene glycol (50 mL), transferred into an autoclave, and subjected to solvothermal treatment at 180 °C for 24 h. The obtained sample was washed by anhydrous ethanol and deionized water several times, and dried at room temperature.

#### 3.2.2. Preparation of m-SiO_2_/Fe_3_O_4_

Fe_3_O_4_ spheres (0.1 g) were dispersed in deionized water (100 mL), then aqueous ammonia (3 mL) and TEOS (0.3 mL) were added, and the mixture was stirred mechanically for 1 h. The solid was collected by a magnet, washed with deionized water and ethanol, and dried at room temperature.

The solid product mentioned above (0.1 g) was mixed with deionized water (100 mL). CTAB (0.2 g) aqueous ammonia (3 mL) was added, and the mixture was stirred at room temperature for 30 min. TEOS (2 mL) was then added, and the mixture was continuously stirred for 8 h. The obtained product was washed with anhydrous ethanol and water several times, dried at 100 °C for 4 h, and calcined at 500 °C for 4 h. The resulting material is denoted as m-SiO_2_/Fe_3_O_4_.

#### 3.2.3. Preparation of Au/m-SiO_2_/Fe_3_O_4_ and Au/SiO_2_ Catalysts

To prepare gold colloids, NaBH_4_ (19 mg) was dissolved in deionized water (5 g), and cooled in a refrigerator (5 °C). HAuCl_4_·4H_2_O (20 mg) and PVP (10 mg) were dissolved in deionized water (95 g). After stirring the mixture for 30 min, the NaBH_4_ solution was injected. After stirring for 1 h, gold colloid solution (3 mL, containing 0.3 mg Au) were mixed with m-SiO_2_/Fe_3_O_4_ spheres (0.1 g) or a conventional SiO_2_ support (surface area 176 m^2^/g), subjected to ultrasonic treatment for 10 min, and left standing for 10 min. The mixture was subjected to centrifugation after adding some water, and the obtained product was dried at room temperature. The theoretical gold content was 0.3 wt%, whereas the actual gold contents measure by ICP were 0.28 wt% and 0.30 wt%, respectively.

### 3.3. Characterization

XRD patterns were collected on a Bruker AXS D8 Advance diffractometer using Cu Kα radiation. SEM images were obtained by a FEI Quanta FEG 250 field-emission scanning electron microscope operated at 20 kV. TEM images were obtained by a Tecnai F20 transmission electron microscope operated at 200 kV. The surface areas were measured on an ASAP-2020 M analyzer. Magnetic properties were measured using MPMS SQUID VEM system. ICP analysis of gold content was conducted using Perkin-Elmer Optima 2100 instrument.

### 3.4. Catalytic Reduction of p-Nitrophenol with NaBH_4_

Catalyst (10 mg) was dispersed in deionized water (50 mL) with the aid of ultrasonic treatment. 300 mM NaBH_4_ solution (1 mL), 3 mM *p*-nitrophenol solution (0.05 mL), and water (1 mL) containing catalyst (mentioned above, 0.2 mg) was added into a cuvette, and the mixture was subjected to absorption measurement at 400 nm every 2 s, by using an UV-Vis-3300 spectrometer (Shanghai Meipuda, Shanghai, China).

## 4. Conclusions

An Au/m-SiO_2_/Fe_3_O_4_ catalyst was prepared by using magnetic Fe_3_O_4_ spheres as a core and support, followed by coating the core with a porous SiO_2_ shell via a “soft-templating” approach and deposition of gold nanoparticles. The catalyst showed higher activity than Au/SiO_2_ in the catalytic reduction of *p*-nitrophenol with NaBH_4_. It also showed good thermal stability. The mesoporous SiO_2_ coatings play an important role in stabilizing supported gold nanoparticles.

## Supplementary Materials

Supplementary materials can be accessed at: http://www.mdpi.com/1420-3049/18/11/14258/s1.
