# Supplementary material for: Gold Nanoparticles on Mesoporous SiO2-Coated Magnetic Fe3O4 Spheres: A Magnetically Separatable Catalyst with Good Thermal Stability
Source: Molecules. 2013 Nov 18;18(11):14258–67. doi: 10.3390/molecules181114258 (PMC6270561; doi:10.3390/molecules181114258)
Supplement: Supplementary file 1 [file molecules-18-14258-s001.pdf]

Supplementary Materials

Figure S1. The size distributuion of Fe_3O_4 spheres. The average particle size is 273 nm.

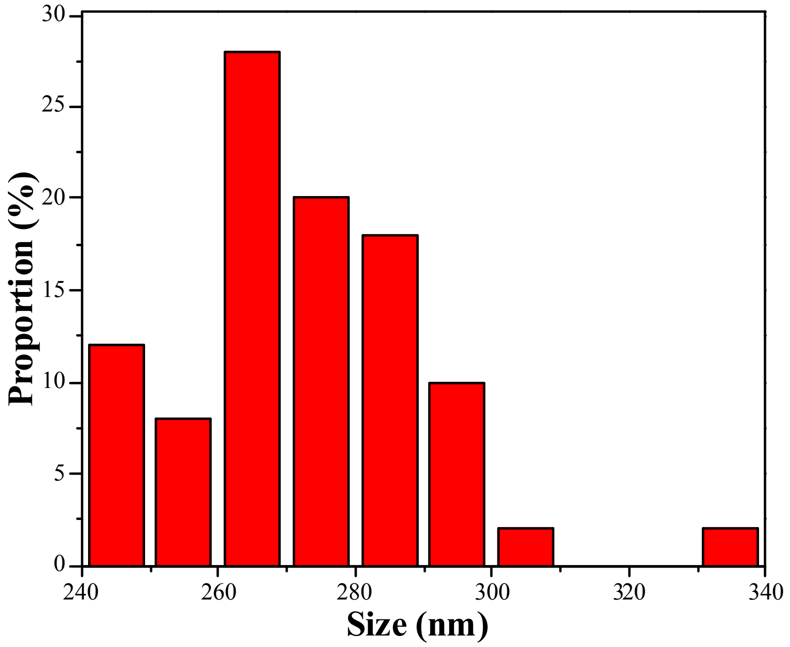

Figure S2. TEM images of Fe_3O_4 spheres coated by a thin layer of SiO_2.

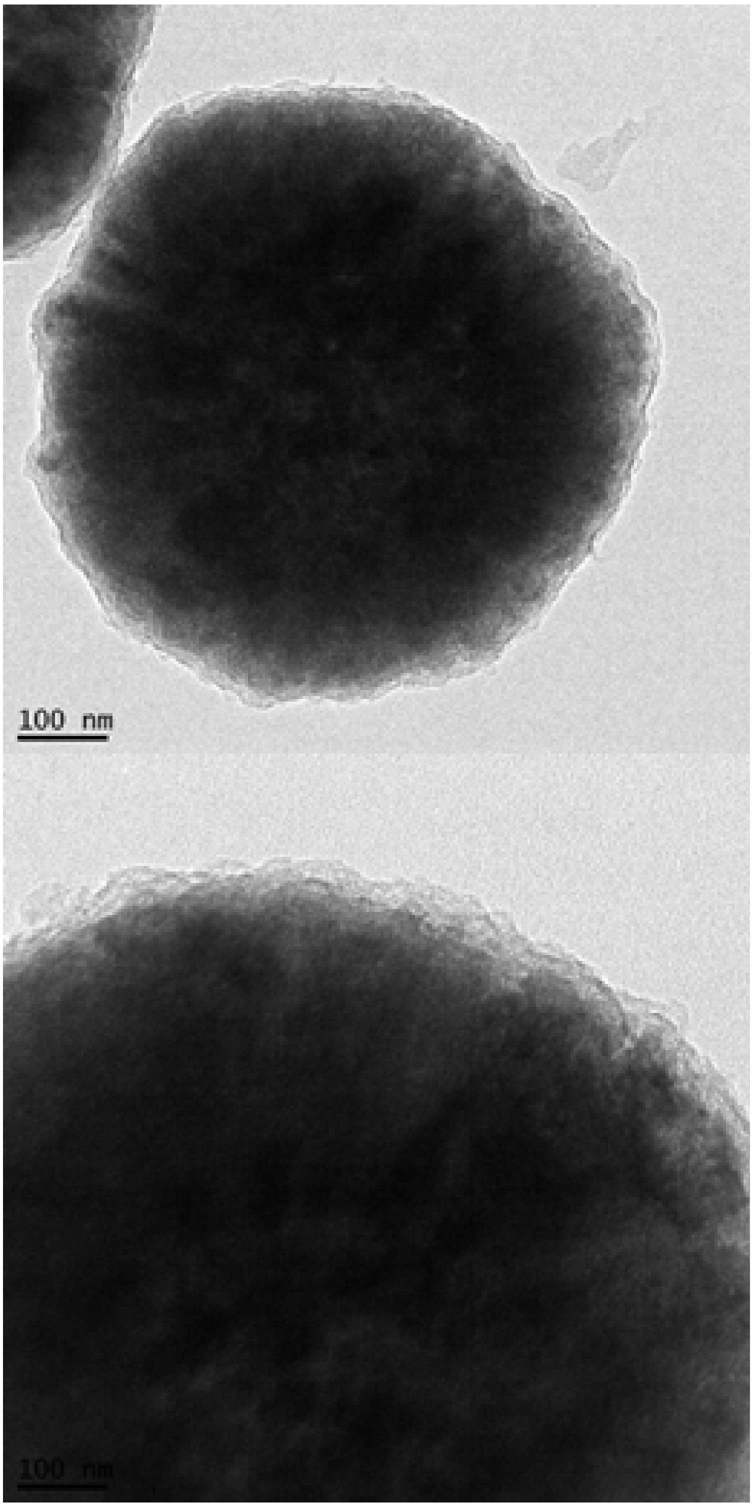

Figure S3. Additional TEM images of m-SiO_2/Fe_3O_4.

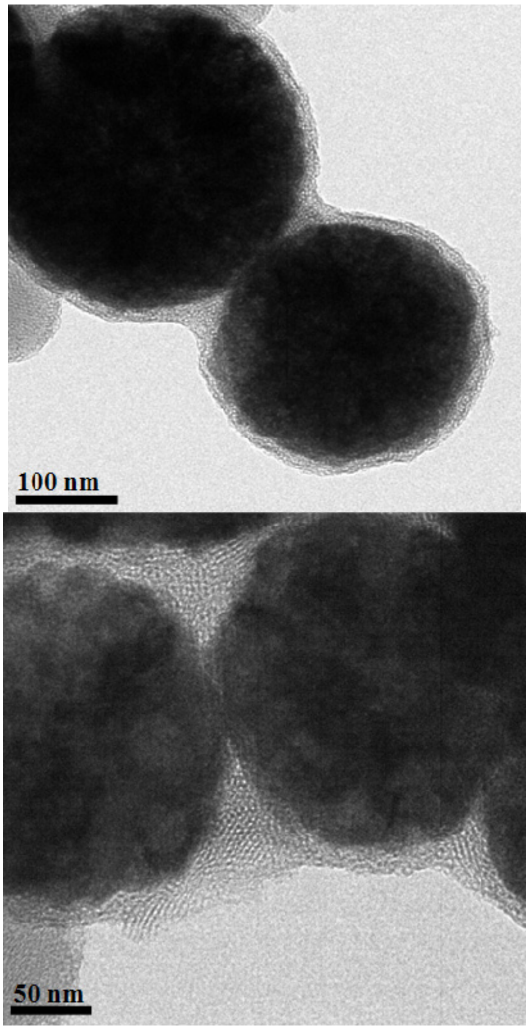

Figure S4. Gold particle size distributions of as-synthesized Au/m-SiO_2/Fe_3O_4 (**a**), Au/m-SiO_2/Fe_3O_4 calcined at 500 °C (**b**), as-synthesized Au/SiO_2 (**c**) and Au/SiO_2 calcined at 500 °C (**d**).

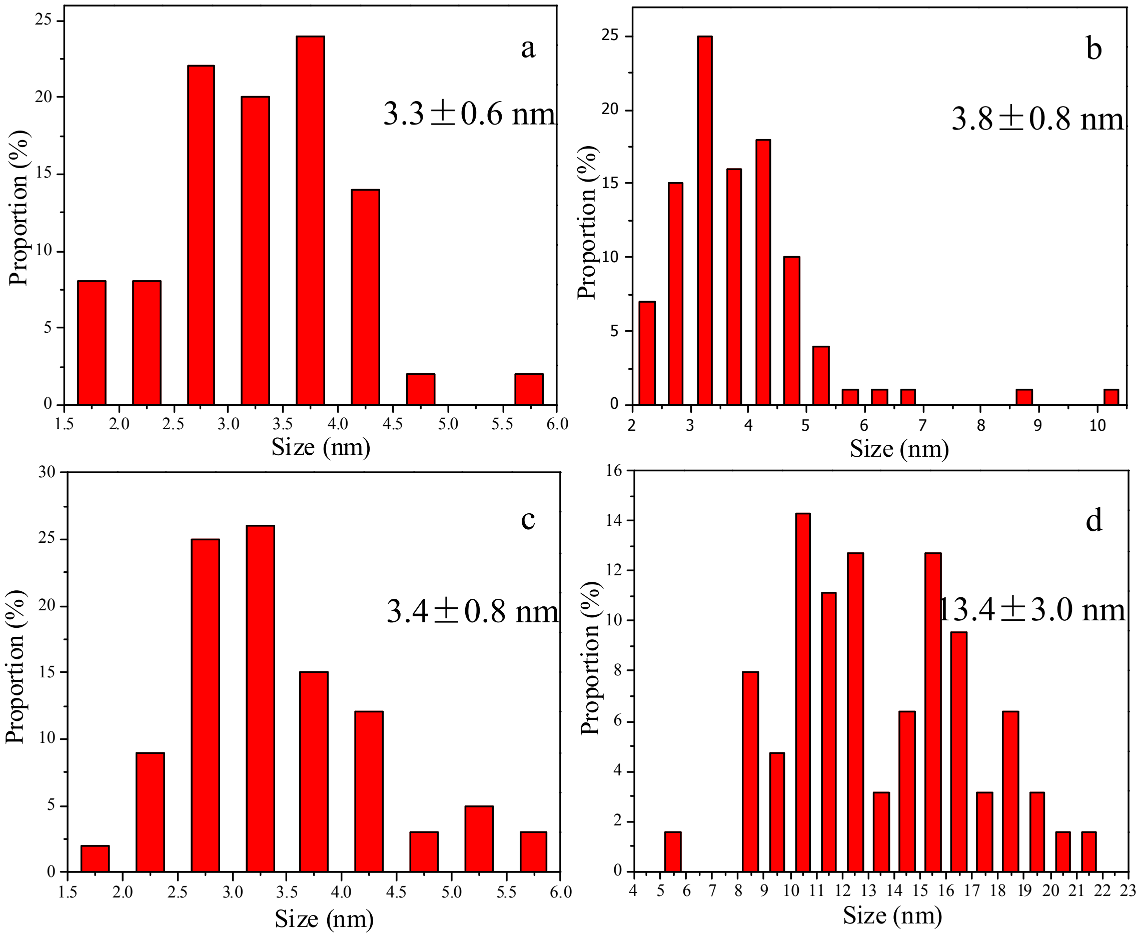

Figure S5. Plots of the Ln(C_t/C_0) versus time for Au/m-SiO_2/Fe_3O_4 and as-synthesized Au/SiO_2 (**a**); Plots of Ln(C_t/C_0) versus time for Au/m-SiO_2/Fe_3O_4 calcined at 500 °C and Au/SiO_2 calcined at 500 °C (**b**).

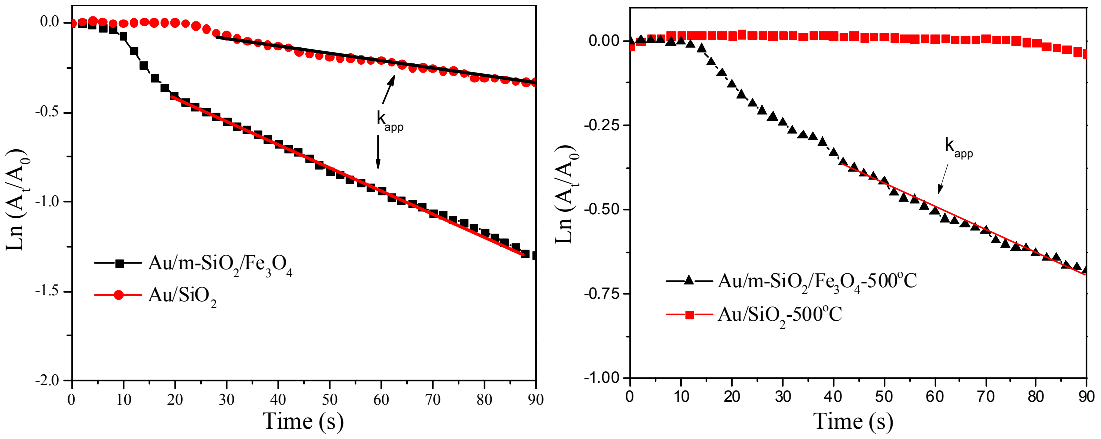

Table S1. Gold loading and catalytic activity of catalysts.

**Sample**	**Au Loading (wt%)** [a]	**Conversion (%)** [b]	**K_{app} (s^{-1})**
Au/m-SiO_2/Fe_3O_4	0.28	72.5	1.26×10^{-2}
Au/SiO_2	0.30	28.2	0.46×10^{-2}
Au/m-SiO_2/Fe_3O_4-500℃	0.28	49.4	0.67×10^{-2}
Au/SiO_2-500℃	0.30	4.5	0

[a] Gold loading measured by ICP; [b] Conversion at 90 s of reaction.
